# Metabolic modulation of brain network dynamics during alcohol withdrawal

**DOI:** 10.64898/2026.10.07.26364946

**Published:** 2026-10-11

**Authors:** Julia K Brynildsen, Ehsan Shokri-Kojori, Corinde E Wiers, Nora D Volkow, Dani S Bassett

## Abstract

Chronic heavy alcohol use shifts brain metabolism from glucose toward acetate utilization. Sudden decreases in acetate following alcohol cessation in individuals with alcohol use disorder (AUD) may create an energetic imbalance that contributes to craving and withdrawal. A recent randomized study found that, compared with a standard American (SA) diet, a ketogenic diet (KD) during alcohol detoxification reduced withdrawal severity and craving and increased ketones and glutamate in the dorsal anterior cingulate cortex. These findings suggest that ketones may support metabolic adaptation during alcohol withdrawal, but the effects of altered brain metabolism on neural dynamics and alcohol cue reactivity remain unknown. Here, we used a control theoretic approach to test whether shifts in cerebral energy substrate utilization influence large-scale network dynamics through altered information flow. We analyzed fMRI cue-reactivity scans collected after one, two, and three weeks of inpatient treatment during presentation of alcohol, food, and neutral cues. Whole-brain effective connectivity was estimated using Shannon transfer entropy applied to whole brain atlas-aligned fMRI time series. Assuming neural activity flow along effective connections, we identified distinct shifts in the state-transition landscape in participants receiving the KD compared with the SA diet. Increases in serum β-hydroxybutyrate (BHB) were associated with changes in state-transition landscape organization, which were in turn associated with reduced cue-induced alcohol craving. These findings link metabolic adaptation to large-scale neural dynamics and clinically relevant behavior and suggest that cerebral energy substrate availability may shape behavior by altering the organization of large-scale neural dynamics.

## Full Text Availability

The license terms selected by the author(s) for this preprint version do not permit archiving in PMC. The full text is available from the preprint server.

